# Geographical Distribution and Trends Analysis of Osteopathic General Surgery Residents

**DOI:** 10.7759/cureus.70641

**Published:** 2024-10-01

**Authors:** Michael D Ernst, Vincent S Alexander, Ryan Wong, Nicholas Berg, Hayden Roberts, Andrew D Vogel, J. Bracken Burns, Kristen Conrad-Schnetz

**Affiliations:** 1 Department of Research, Alabama College of Osteopathic Medicine, Dothan, USA; 2 Medicine, Nova Southeastern University Dr. Kiran C. Patel College of Osteopathic Medicine, Fort Lauderdale, USA; 3 Department of Research, Rocky Vista University College of Osteopathic Medicine, Ivins, USA; 4 Division of Research, New York Institute of Technology College of Osteopathic Medicine, Arkansas, USA; 5 Department of Surgery, East Tennessee State University, Johnson City, USA; 6 Department of Surgery, Cleveland Clinic South Pointe Hospital, Cleveland, USA

**Keywords:** general surgery, geography, osteopathic, region, residency match, state

## Abstract

In recent years, the number of Doctor of Osteopathic Medicine (DO) residents entering general surgery has increased. As DOs continue to solidify their role within the surgical domain, understanding their distribution, preferences, and the dynamics of their integration into residency programs becomes crucial for general surgery applicants. Publicly available data were gathered for each DO general surgery resident from residency programs across the nation, including details such as post-graduate year, degrees held, and residency program location. A comprehensive cross-sectional analysis was conducted to determine the geographical distribution and match trends of DO residents in these residency programs. Analysis revealed a significant rise in the number of DOs entering general surgery residencies, from 153 DO trainees beginning their residency training in 2019 to 274 DO trainees beginning their residency training in 2024. This upward trend indicates a growing presence of DOs in surgical practice. Examination of the geographical locations of programs for which DO applicants have matched showed variations nationwide. This analysis allows for a deeper understanding of the evolving match trends for DOs in surgery. It highlights the importance of addressing disparities in access to surgical training opportunities for osteopathic physicians nationwide.

## Introduction

There has been a significant change in the landscape of general surgery education in the United States, with an increasing number of osteopathic physicians (DO) applying and successfully matching into the field [1]. While the rise in DO general surgery applicants is noteworthy, there is a lack of comprehensive data on the geographic spread and yearly trends of where DO general surgery residents are matching. Understanding these patterns is vital for making informed decisions for DO applicants.

Notably, for 2019, the National Resident Matching Program (NRMP) reported that 143 DO seniors had been matched into categorical general surgery positions [2]. However, publicly available NRMP data captures a picture of only the matched class and does not provide a longitudinal data analysis for postgraduate years 2-5. By examining the geographic distribution and trends among DO general surgery residents, this study aims to identify areas where there is greater representation of DO surgical trainees and the trends in their regional location of post-graduate training.

## Materials and methods

All ACGME-approved categorical general surgery residency programs were identified from the official ACGME website source in August of 2023 [2]. Webpages associated with each residency program were then identified to extrapolate the following resident and program characteristics: resident’s post-graduate year, degree, and program location. Residency program websites were defined as up-to-date by either explicitly indicating the current 2023-2024 academic year or cross-referenced with publicly available resident data online. This included reporting a resident’s graduation year or a professional profile via LinkedIn, Doximity, or X (formerly known as Twitter). All programs without up-to-date websites were excluded from the analysis. Additionally, the number of active DO schools each year was determined between 2015 and 2024 using the publicly available American Osteopathic Association’s Osteopathic Medical Profession Report, which is published yearly [3]. A cross-sectional analysis of DO match trends and geographical distribution within general surgery residency programs across the United States was conducted. The geographical heatmap was generated using Google Sheets (November 2023, Mountainview, CA). Descriptive statistics and regression analysis to look at the number of DO general surgery residents over the past six years and changes in the number of DO schools were performed using GraphPad Prism version 10.1.2 for Windows (GraphPad Software, Inc., San Diego, CA). A p-value of <0.05 was considered significant.

## Results

**Figure 1:**
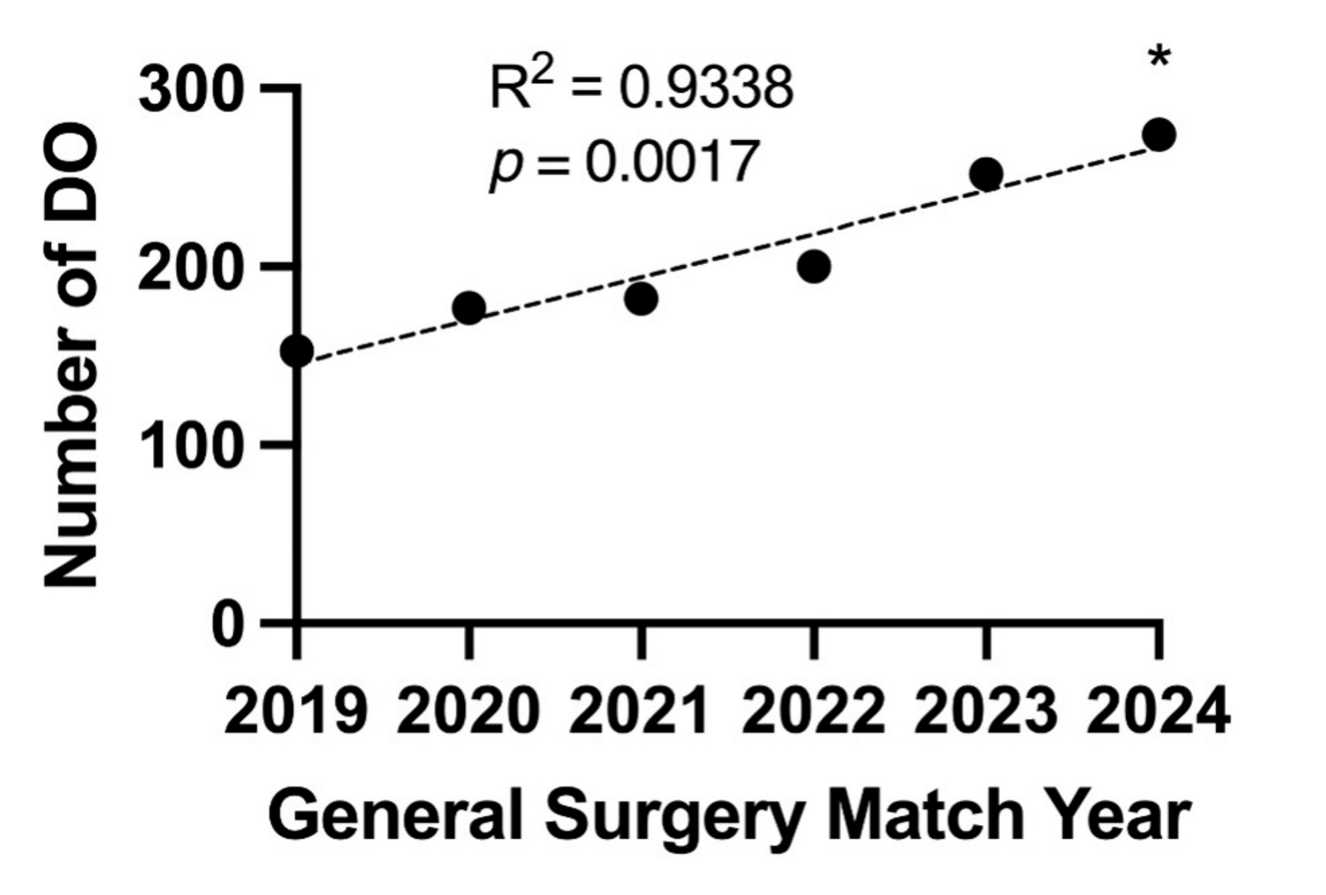
Categorical general surgery match trends from 2019 to 2024. Number of DO applicants that matched each year. *Denotes 2024 data extrapolated from NRMP match results.

**Figure 2:**
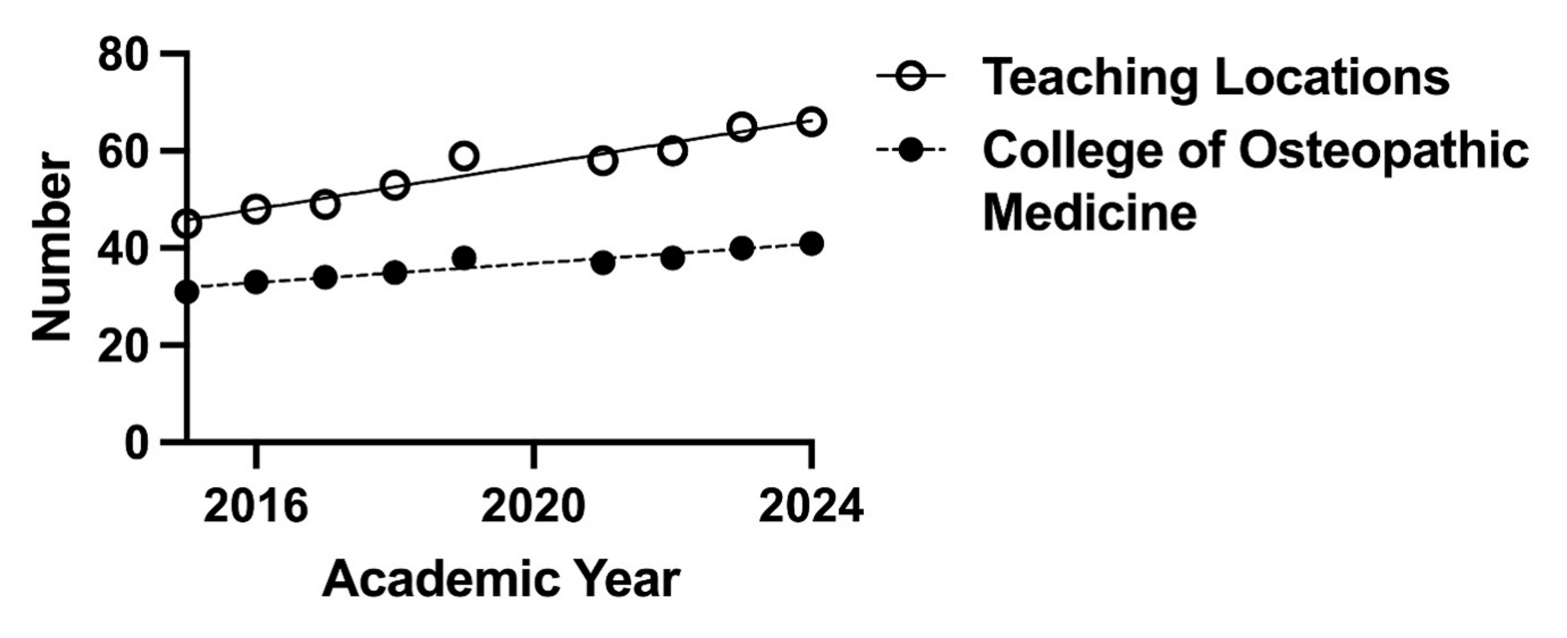
Number of Colleges of Osteopathic Medicine and their associated teaching locations between 2015 and 2024.

There were 270 up-to-date general surgery residency program websites, and 7,476 residents were identified for the 2023-2024 academic year. The majority (n = 6,511, 87.09%) held an MD degree. Nine hundred sixty-five residents (12.91%) were found to hold a DO degree from a total of 185 general surgery programs (68.5%). The number of DOs that matched into general surgery has significantly increased from 143 in 2019 to 274 in 2024 (R2 = 0.9338, p = 0.0017), as seen in Figure 1. Our data collection found that 153 DO residents had actually started their categorical general surgery residency training in 2019. The discrepancy between the number of matched categorical general surgery DO candidates (143) in 2019 and the number of DO residents who began categorical general surgery residency in 2019 (153) may be due to a number of post-match factors. The number of colleges of osteopathic medicine and their associated teaching locations has also increased over the past nine years, as seen in Figure 2.

**Figure 3:**
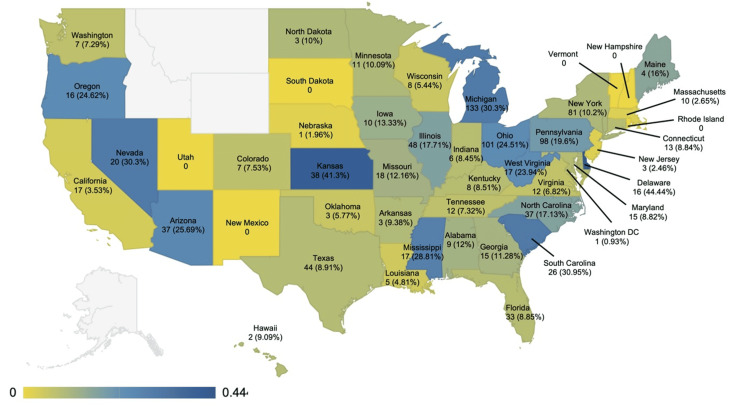
Geographical heatmap of the ratio of DO general surgery categorical residents for the 2023-2024 academic year by state. Image credits: Ryan Wong. White indicates no data. Values are labeled as n (%).

The geographical distribution of DO general surgery residents is depicted in Figure 3. Delaware had the highest proportion of DO general surgery residents (n = 16, 44.44%). Kansas (n = 38, 41.3%) had the second highest, and South Carolina (n = 26, 30.95%) had the third highest. When looking at the absolute number of DO general surgery residents by state, Michigan had the greatest (n = 133, 30.3%). Ohio had the second highest (n = 101, 24.51%), and Pennsylvania had the third highest (n = 98, 19.6%). Six states with general surgery residency programs had no DO general surgery residents, including New Hampshire, New Mexico, Rhode Island, South Dakota, Utah, and Vermont. Several states without general surgery residency programs were incompatible for analysis, including Alaska, Idaho, Montana, and Wyoming. 

**Figure 4:**
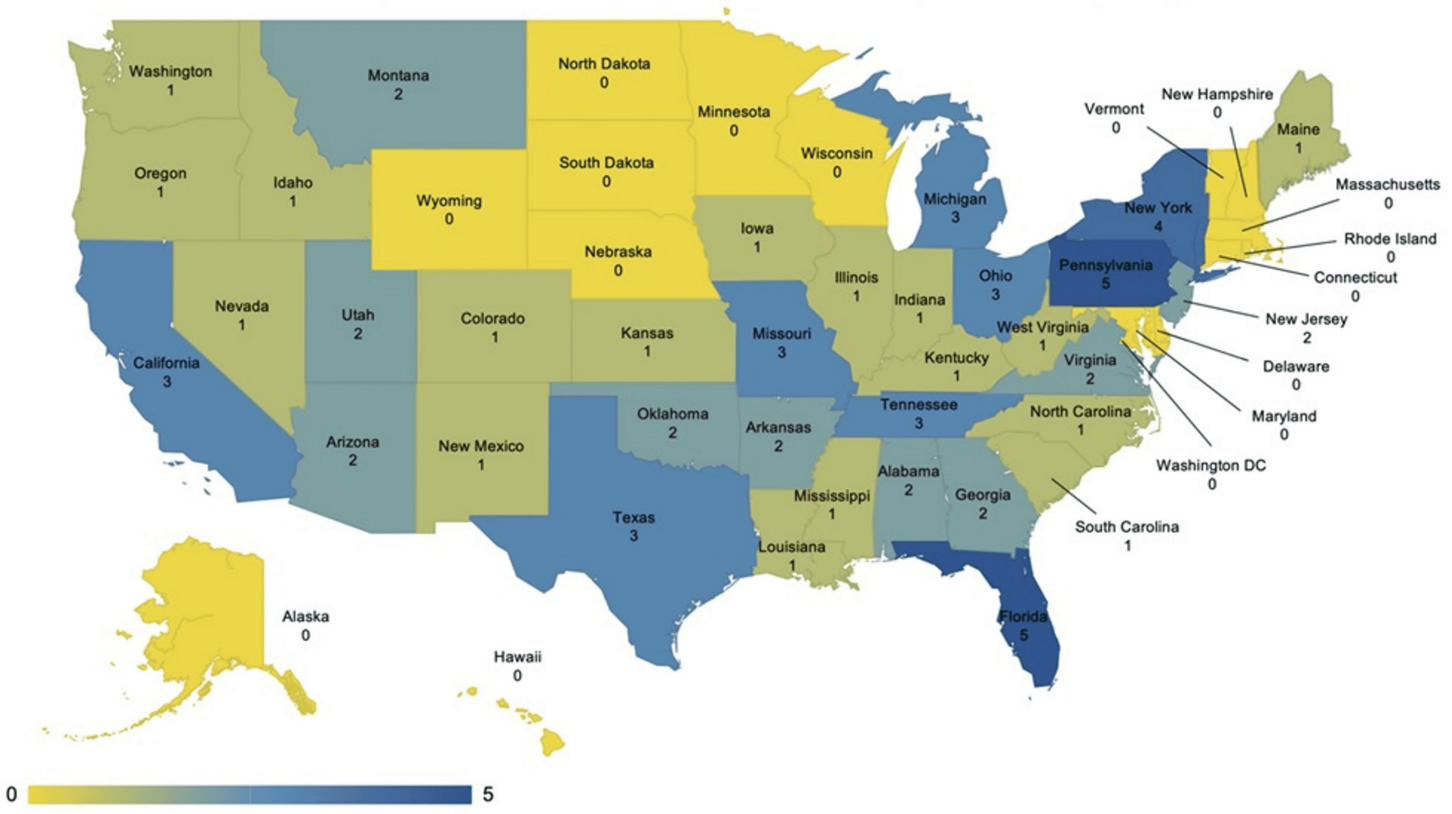
Geographical heatmap of college of osteopathic medicine’s associated teaching locations in 2024. Image Credits: Ryan Wong.

The geographical distribution of colleges of osteopathic medicine in 2024 is depicted in Figure 4. The three states with the highest number of DO general surgery residents corresponded with those having three to five COM teaching locations. California, Texas, and Florida, which have three to five COM teaching locations, had low proportions of DO general surgery residents. The New England region, which has a low number of COM teaching locations, also had low numbers of DO general surgery residents.

## Discussion

Over the past five years, there has been a rise in the number of DOs matching into ACGME-accredited categorical general surgery residency positions from 153 in 2019 to 274 in 2024 [4,5]. Meanwhile, the number of DO categorical general surgery residency applicants to ACGME-accredited categorical general surgery residency positions only rose from 525 in 2019 to 569 in 2024. The increase in the DO categorical general surgery match rate into ACGME-accredited categorical general surgery positions from 29.1% in 2019 to 48.2% in 2024 highlights the increasing match success of osteopathic medical graduates interested in pursuing careers in general surgery [5]. However, the number of allopathic medical students who matched into a categorical general surgery residency position has consistently stayed around 1050 (± 20) matches from 2019-2024 [4]. The increasing number of osteopathic students matching into general surgery suggests increasing recognition and acceptance of osteopathic physicians in the broader medical community, particularly in fields traditionally made up of their allopathic counterparts. This shift may reflect the evolving landscape of medical education and practice, where the distinctions between DOs and MDs are becoming less pronounced and greater focus is placed on the individual qualities and intrinsic worth of the applicants themselves through holistic screening processes. This change suggests that selection committees are placing greater emphasis on the diverse skills, perspectives, and personal commitments each applicant brings to the table beyond the distinctions of their medical education degree.

Determining where osteopathic applicants have matched geographically elucidates potential disparities between states nationwide. The data collected during this study revealed geographical trends consistent with a previous study that identified the prevalence of DO general surgery residents training in each state [6]. States, including Delaware, Kansas, and South Carolina, have a higher percentage of DO general surgery residents compared to others. Conversely, states such as Michigan, Ohio, and Pennsylvania have a greater overall absolute number of DO general surgery residents. Interestingly, there is a complete lack of any general surgery-matched applicant training in Alaska, Idaho, Montana, and Wyoming. There are also still states with general surgery residencies that lack DO representation. 

Some of the states that were identified through our analysis as having the most osteopathic medical schools when compared with other states had some of the lowest percentages of DO trainees within general surgery residency programs. The data from our study, which in general showed a positive correlation between the number of COM teaching locations and the number of DO general surgery residents within a state, suggests a potential area of focus moving forward for improving general surgery training opportunities for DOs. The New England region, which has a low number of COM teaching locations, also had low numbers of DO general surgery residents. In contrast, the three states with the highest number of DO general surgery residents corresponded with those having three to five COM teaching locations. However, some notable exceptions to this positive correlation include California, Texas and Florida, which each have three to five COM teaching locations but have relatively low proportions of DO general surgery residents when compared with other states. These data suggest that there may be other factors limiting the number of DO general surgery trainees in these states, such as California, Texas, and Florida. While further studies are needed to determine any causality, this positive correlation indicates an important area for further analysis and the potential for improving equity for DOs pursuing general surgery in the match process by increasing the number of COM teaching locations throughout the country.

While there are many potential factors contributing to these discrepancies amongst these states, multiple factors have already been identified in previous studies as significant determining factors in DO general surgery match results. Some of these factors include details such as regional preferences among osteopathic medical graduates as well as existing partnerships between osteopathic medical schools and general surgery residency programs [7,8]. Access to mentorship opportunities has also been identified to have a significant impact on the match prospects for medical students. A retrospective cross-sectional study of matched and unmatched general surgery applicants from 2018 to 2021 found that 36% of matched general surgery applicants reported some sort of geographic connection to the program where they matched [9]. Data such as this further supports the need for improved integration of osteopathic medical students with general surgery residency programs to improve mentorship opportunities, match outcomes for DOs, and DO representation in the field throughout the country [1,6-8]. Forming collaborations between osteopathic medical schools, healthcare systems, and professional organizations to either initiate new programs or enhance current ones can help further increase the presence of DO trainees in general surgery and support the rising demand for surgical training among DO graduates.

DO faculty representation has also been identified as a significantly associated factor concerning DO matches at general surgery residency programs, with the number of DO faculty being positively correlated with the number of DO matches [10]. A similar correlation was found between the ratio of DO general surgery program directors and the number of DO applicants who match into general surgery for a given year [11]. A study analyzing the effects of the single accreditation system, which was implemented in 2020, found that of 60 former AOA general surgery residency programs, 56% had transitioned to having MD program directors [11]. This decrease in the number of DO general surgery program directors following the single accreditation system is concerning as it may be limiting opportunities for DO trainees to match into general surgery residency programs throughout the country. While the data analyzed in this study show an increase in the DO match rate between 2019 and 2024, the room for increase may be hindered by the decline in DO representation within general surgery program leadership. With the number of DO faculty at a residency program having a significant impact on the match prospects for DO trainees at that program, one way to expand training opportunities for DOs in general surgery is for DO graduates of general surgery residencies to become faculty at institutions that do not currently have DOs and to increase DO representation within general surgery program leadership when possible.

The data from this study identified several rural states, such as Vermont, New Hampshire, North Dakota, South Dakota, and Nebraska, which had limited or no DO representation. While the strategy used to improve DO representation throughout the country may vary depending on the location, one factor that could be focused on concerning improving DO representation in these rural areas is increasing exposure through rural surgical rotations. A meta-analysis of the outcomes of rural surgical rotations during the residency period on attending general surgeon placement found that exposure to rural surgical rotations during residency increased the number of general surgeons practicing in rural areas [12]. With many of these states, which are lacking in DO representation, being comprised of mainly rural areas, improving access to unique training opportunities such as rural surgery rotations may improve representation in these areas specifically over time.

Limitations

Given that our data analysis involved a cross-sectional analysis, there is limited ability to infer causal relationships or longitudinal trends. Another limitation of this study is that data was collected from the websites of general surgery residencies throughout the country. This data collection method, which involved gathering information from the websites of general surgery residencies nationwide, introduces potential variability in data accuracy and completeness due to the dependence on the precision of the information provided on each residency program's website. Future research should focus on identifying factors that influence the representation of DOs in regions with scarce osteopathic presence, especially in rural areas lacking general surgery residents. Additionally, future research should examine the impact of osteopathic medical students' exposure to rural general surgery rotations and the availability of residency programs in these areas, as there is not always a direct correlation between the number of programs and a state's population [13]. Furthermore, assessing the outcomes of the single accreditation system on rural general surgery training for DOs could reveal areas needing improvement to enhance graduate medical education in rural and underserved locations [14,15]. This comprehensive approach could help craft policies and programs that better support the nationwide equitable distribution of surgical training opportunities.

While our data from this study provides some encouraging evidence of greater equity in the match process for DO candidates pursuing a general surgery residency position, the various other factors previously mentioned point to areas of potential improvement moving forward. Given that many of these factors may affect DO general surgery match results and influence these distribution trends, it is important to understand the geographical trends of matched DO general surgery trainees to compare these trends with these identified contributing factors. These data may provide key insights into how to continue supporting DO representation in general surgery through an increase in DO general surgery residency trainees in these specific areas with a low proportion of DO general surgery residents. Future studies comparing these geographical trends with the aforementioned factors will help identify areas for potential improvement in creating equity in the match process for DO candidates pursuing general surgery as well as the DO representation in the specialty of general surgery and other surgical specialties [16].

## Conclusions

The geographic distribution and patterns of osteopathic general surgery trainees in the US highlight the rising number of osteopathic doctors entering general surgery training programs, showcasing the increasing presence of DOs in the surgical field. The improvement in DO representation in the surgical field over the past five years is encouraging. With the continued growth of osteopathic surgery applicants matching every year, there is hope that continued growth will increase diversity and enrich the pool of surgical expertise nationwide. This trend highlights the growing acceptance and integration of osteopathic physicians in mainstream medical practices and suggests a promising shift towards a more encompassing healthcare system. This burgeoning inclusivity and collaboration signal a brighter future for healthcare, where the fusion of varied medical philosophies and practices enhances the medical community as a whole.
